# Molecular basis of the interaction between gating modifier spider toxins and the voltage sensor of voltage-gated ion channels

**DOI:** 10.1038/srep34333

**Published:** 2016-09-28

**Authors:** Carus H. Y. Lau, Glenn F. King, Mehdi Mobli

**Affiliations:** 1 Institute for Molecular Bioscience, The University of Queensland, St. Lucia, QLD 4072, Australia; 2 Centre for Advanced Imaging, The University of Queensland, St. Lucia, QLD 4072, Australia

## Abstract

Voltage-sensor domains (VSDs) are modular transmembrane domains of voltage-gated ion channels that respond to changes in membrane potential by undergoing conformational changes that are coupled to gating of the ion-conducting pore. Most spider-venom peptides function as gating modifiers by binding to the VSDs of voltage-gated channels and trapping them in a closed or open state. To understand the molecular basis underlying this mode of action, we used nuclear magnetic resonance to delineate the atomic details of the interaction between the VSD of the voltage-gated potassium channel KvAP and the spider-venom peptide VSTx1. Our data reveal that the toxin interacts with residues in an aqueous cleft formed between the extracellular S1-S2 and S3-S4 loops of the VSD whilst maintaining lipid interactions in the gaps formed between the S1-S4 and S2-S3 helices. The resulting network of interactions increases the energetic barrier to the conformational changes required for channel gating, and we propose that this is the mechanism by which gating modifier toxins inhibit voltage-gated ion channels.

Voltage-gated ion channels (VGICs) are large membrane proteins that conduct ions down their concentration gradient in response to changes in membrane potential. They play important functional roles in a wide range of organisms, including archaea, bacteria, and all animal phyla^1^. VGICs are typically either homo- or hetero-tetramers (e.g., voltage-gated potassium (K_V_) channels) or monomers containing four homologous but non-identical domains (e.g. mammalian voltage-gated sodium (Na_V_) channels). Each subunit or domain is comprised of six transmembrane helices denoted S1-S6. The C-terminal S5 and S6 helices and the intervening re-entrant loop form the central pore at the interface between subunits, while each of the S1-S4 elements forms a voltage-sensing domain (VSD) that enables the channel to sense and respond to changes in transmembrane voltage. The S4 helix, which contains the gating charges (Arg/Lys residues), and a portion of the S3 helix form a voltage-sensing paddle^2^ whose movement in response to changes in the transmembrane voltage is coupled to opening of the channel pore^2^,^3^,^4^.

Ion channels are the primary targets of insecticides^5^,^6^ as well as the principal therapeutic target for ~13% of extant drugs, making them the second largest drug class after G-protein coupled receptors^7^. Consequently, there are numerous ongoing efforts to develop selective modulators of both ligand and voltage-gated ion channels^8^. The venoms of invertebrate predators such as spiders, scorpions, and cone snails have evolved to target neuronal ion channels and receptors, and consequently they are the best natural source of ion channel modulators^9^,^10^,^11^,^12^. While many animal toxins are pore blockers that prevent entry of ions into the pore of VGICs, arachnid venom peptides are unusual in that they are mostly gating modifiers that specifically bind to the VSD in order to trap the channel in either the closed (resting) or open (activated/inactivated) state^11^. Consequently, these peptides can serve as leads for drug and insecticide development whilst also being useful pharmacological tools for trapping VGICs in particular stages of the gating cycle. Numerous studies have been undertaken to understand the structure-activity relationships governing the interaction of these peptides with VGICs but the atomic details remain poorly understood, hindering rational engineering of more selective VGIC modulators^13^,^14^,^15^,^16^,^17^,^18^,^19^,^20^.

Several structural models have recently been proposed to explain how venom peptides interact with VSDs and modulate ion channel function: (i) a docking model, supported primarily by mutagenesis data, suggests that these peptide bind to a cavity formed between the four VSD helices^19^; (ii) a docking model where the peptide binds in a cleft between helix S1 and S4 supported by ambiguous NMR restraints^13^; and (iii) a docking model supported by fluorescence data indicating that the peptide binds residues on the S3 helix^21^. In parallel to these peptide-binding studies it was recently demonstrated, using X-ray crystallography that a small-molecule antagonist of Na_V_1.7 binds into a cleft formed between the four helices of the domain IV VSD, accessing the cavity from a gap formed between the S2 and S3 helices. It is, however, unclear whether gating-modifier venom peptides bind to VSDs in a similar manner^8^.

VSDs are structurally autonomous domains, as evidenced by the fact that the NMR-derived structure of the isolated VSD from the archaebacterial Kv channel KvAP (VSD_K_)^22^,^23^ is essentially identical to its conformation in the crystal structure of the full-length channel^24^,^25^. Moreover, VSDs can be found in completely different structural contexts in non-VGIC proteins such as voltage-sensing phosphatases^26^. Here we took advantage of the portability of VSDs to apply NMR spectroscopy to delineate the molecular basis by which gating modifier toxins immobilise VSDs, using the interaction between KvAP and the spider-venom peptide VSTx1 as a model system^27^. The KvAP-VSTx1 interaction has been functionally well characterised and the mechanism of channel inhibition is consistent with venom peptides that upon membrane depolarisation trap the channel in an inactivated state and prevent the conformational transition back to the resting state for subsequent channel opening^28^.

In contrast to previous peptide-VSD studies our data provide atomic resolution information about the VSTx1–VSD_K_ interaction. NMR data and complementary mutagenesis studies reveal that VSTx1 interacts with residues within the aqueous cavity formed on the extracellular surface of the channel between the S1-S2 and S3-S4 loops. A docking model based on experimental constraints reveals a binding mode that agrees remarkably well with previous mutagenesis data on a related gating modifier peptide–Na_V_ complex^14^, suggesting a conserved binding mode where the peptide binds to acidic residues on the diagonally opposed S1 and S3 helices. In this model the lipophilic surface of the peptide is positioned against the lipid-facing gaps formed between the S2-S3 and S1-S4 helices. Our combined data indicate that the extensive network of peptide-channel-lipid interactions provides the increased energetic barrier required to inhibit the conformational change necessary for channel gating, and we propose that this is the primary mechanism by which gating modifier toxins inhibit VGICs. These findings provide a structural platform for the rational design of therapeutics and insecticides that target ion channel VSDs.

## Results

### Structure of VSTx1

**Figure 1:**
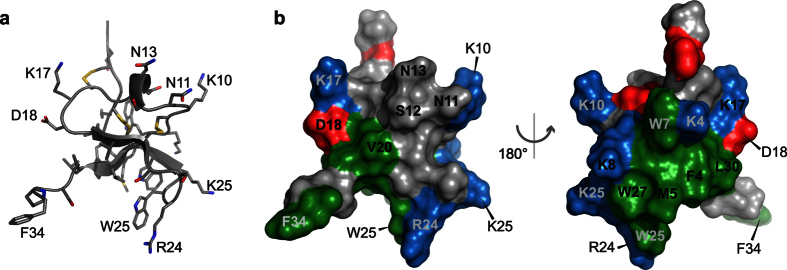
Structure of VSTx1. (**a**) Solution structure of VSTx1 determined using heteronuclear NMR (PDB code 2N1N). **(b)** Molecular surface of VSTx1 highlighting the hydrophobic patch (green) surrounded by five cationic residues (blue). Anionic residues are shown in red.

Recombinant VSTx1 was produced as an MBP fusion protein using an *E. coli* periplasmic expression system designed for production of disulfide-rich peptides^29^. Cleavage of the fusion protein yielded the VSTx1 peptide with an additional serine residue at the N-terminus. This recombinant version is similar to that prepared previously by Ruta *et al*. where the N-terminus contained an additional GS sequence^27^. This N-terminally extended recombinant peptide was reported to inhibit KvAP with similar potency to the WT peptide. For ease of comparison with existing literature the WT numbering of VSTx1 is used throughout this report. Recombinant expression facilitated isotope labeling of the toxin for subsequent NMR studies. A structure of VSTx1 has previously been reported based on homonuclear NMR data (PDB code 1S6X)^30^. Here, we used heteronuclear NMR at ultra-high field (900 MHz) to determine the structure of recombinant VSTx1, which when compared to the existing VSTx1 structure confirmed correct folding of the peptide^30^. Disulfide bond connectivities were confirmed by 3D NOESY spectra as previously described^31^. Structure calculations were based on 694 interproton distance restraints derived from NOESY spectra, 54 dihedral angle restraints from TALOS-N chemical shift analysis^32^, 14 hydrogen-bond restraints derived from a long-range HNCO experiment, and nine disulfide-bond constraints, yielding a total of 762 constraints. Table 1 summarises these restraints and the statistics obtained for the calculated ensemble of structures. The new ensemble (Fig. 1a) is more precisely defined than the previously reported structure, with backbone and heavy-atom with root-mean-squared deviation (RMSD) values of 0.03 Å and 0.54 Å, respectively (cf 1S6X, 0.44 Å and 1.08 Å for backbone and heavy atoms respectively). The assigned chemical shifts and 3D structure have been deposited in the relevant databases (BMRB accession number 25568 and PDB code 2N1N).

The structure of VSTx1 reveals 10 hydrophobic residues on the toxin surface, most of which are clustered together in a hydrophobic patch surrounded by five basic residues (Lys4, Lys8, Lys17, Arg24, and Lys26) (Fig. 1b). The structure explains the unusual chemical shifts observed for some protons. The Hα of Met6 is sandwiched between Phe5 and Trp26 and the unusual upfield chemical shift of this proton (2.97 ppm, compared with an average shift for this proton of 4.40 ppm) is due to ring currents from the aromatic rings of these two residues (Fig. 1a). The amide proton of Lys4 also has an unusual upfield chemical shift due to its proximity to the indole ring of Trp7.

### The hydrophobic patch on VSTx1 mediates its interaction with lipid membranes

Structural studies of VSD_K_ require this membrane domain to be solubilised in detergent micelles. For this purpose we used 1,2-diheptanoyl-*sn*-glycero-3-phosphocholine (DHPC) micelles as employed in previous NMR characterisation of VSD_K_^22^. This phospholipid micelle approximates the chemical environment of phospholipid bilayers^22^. Considering that VSTx1 was previously reported to partition into liposomes^30^,^33^,^34^, we first examined whether it interacts with DHPC micelles alone using NMR chemical shift mapping (NMR-CSM). This technique, which is commonly used for determining protein-ligand interaction interfaces^35^, relies on the fact that changes in chemical environment induced by non-covalent interactions with binding partners often induces chemical shift perturbations in residues at the interaction interface. Thus, mapping of the changes in chemical shift onto the protein structure provides information about the binding interface and any conformational changes that occur as a result of the interaction.

**Figure 2:**
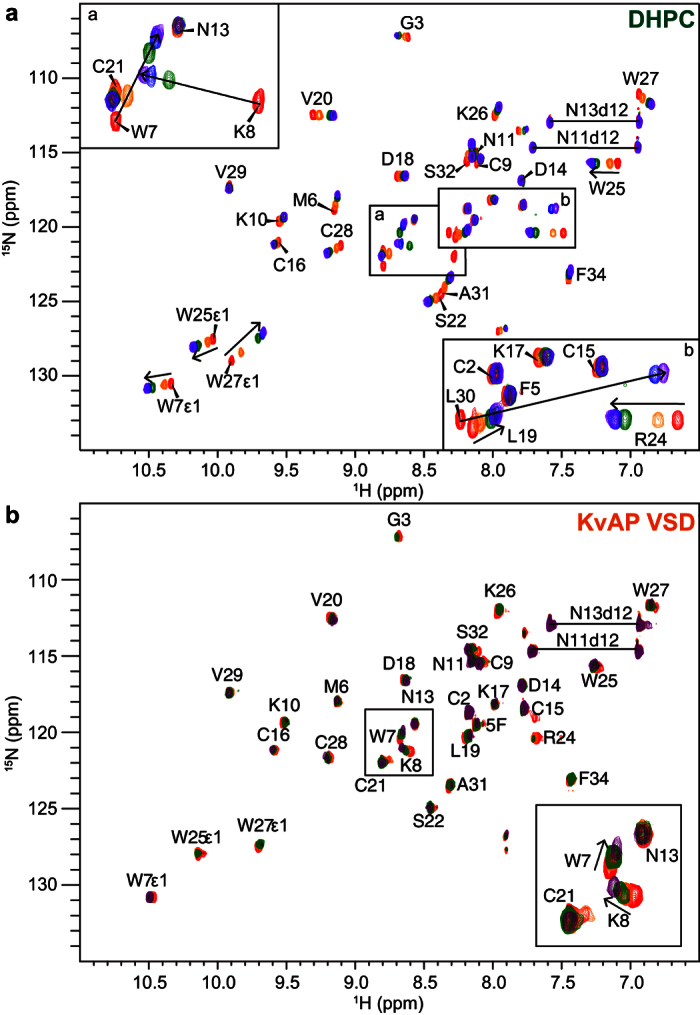
VSTx1 interacts with both DHPC and VSD_K_. (**a**) Overlay of 2D ^1^H-^15^N HSQC spectra of 30 μM ^15^N-labeled VSTx1 acquired under identical conditions in the absence (red) or presence of DHPC (orange: 0.1%, green: 0.2%, blue: 0.5%, purple: 1%). Chemical shifts of resonances from Trp7, Lys8, Trp27, and Leu30 were significantly perturbed by addition of DHPC. (**b**) Overlay of 2D ^1^H-^15^N HSQC spectra of 20 μM ^15^N-labeled VSTx1 acquired under identical conditions in the absence (red) or presence of VSD_K_ (orange: 10 μM, green: 20 μM, purple: 100 μM). The resonances from Arg24 and the sidechain of Trp25 were most severely broadened by addition of VSD_K_.

**Figure 3:**
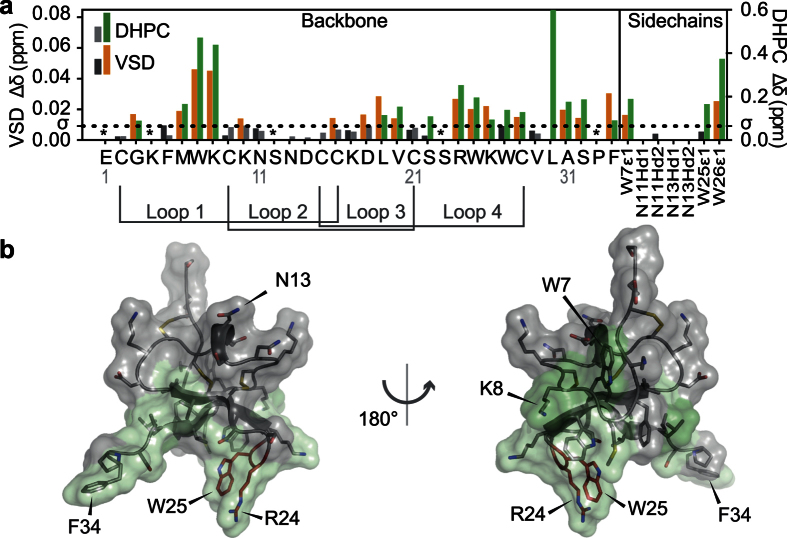
The hydrophobic patch on VSTx1 mediates its interaction with lipids and contains VSD_K_-binding residues. (**a**) Chemical shift perturbations (Δδ) on VSTx1 caused by interaction with DHPC (green) and further enhanced by presence of VSD_K_ (orange). Residues marked with an asterisk were not observable in the NMR spectra. (**b**) Molecular surface of VSTx1 with putative lipid binding residues highlighted in green. Putative VSD_K_-binding residues identified by NMR-CSM are shown with orange side chains. The VSD_K_-binding residues divide two lipophilic segments of the peptide.

In order to identify residues on VSTx1 that interact with detergent micelles, 2D ^1^H-^15^N HSQC spectra of ^15^N-labelled VSTx1 were acquired in the presence of increasing concentrations of DHPC (0–1% w/v) (Fig. 2a). VSTx1 binds only transiently to DHPC micelles (*K*_d_ in the high μM to mM range) giving rise to fast exchange in NMR-CSM experiments, similar to observations in 1-palmitoyl-2-oleoyl-*sn*-glycero-3-phosphocholine (POPC) lipid nanodiscs^36^. A plot of the combined ^1^H/^15^N chemical shift changes (Δδ; see Eqn (1) in Materials and Methods) shows that 16 backbone amides experienced changes in chemical shift higher than the chosen cut-off value of one standard deviation of all perturbations (Fig. 3a). All of these residues are clustered within the hydrophobic patch (Fig. 3b). Chemical shifts for residues Cys9–Asp18 within the intercystine loop on the opposite surface of the toxin were not affected, indicating that the interaction of VSTx1 with DHPC is specifically mediated by the hydrophobic patch, largely consistent with previous studies^13^.

**Figure 4:**
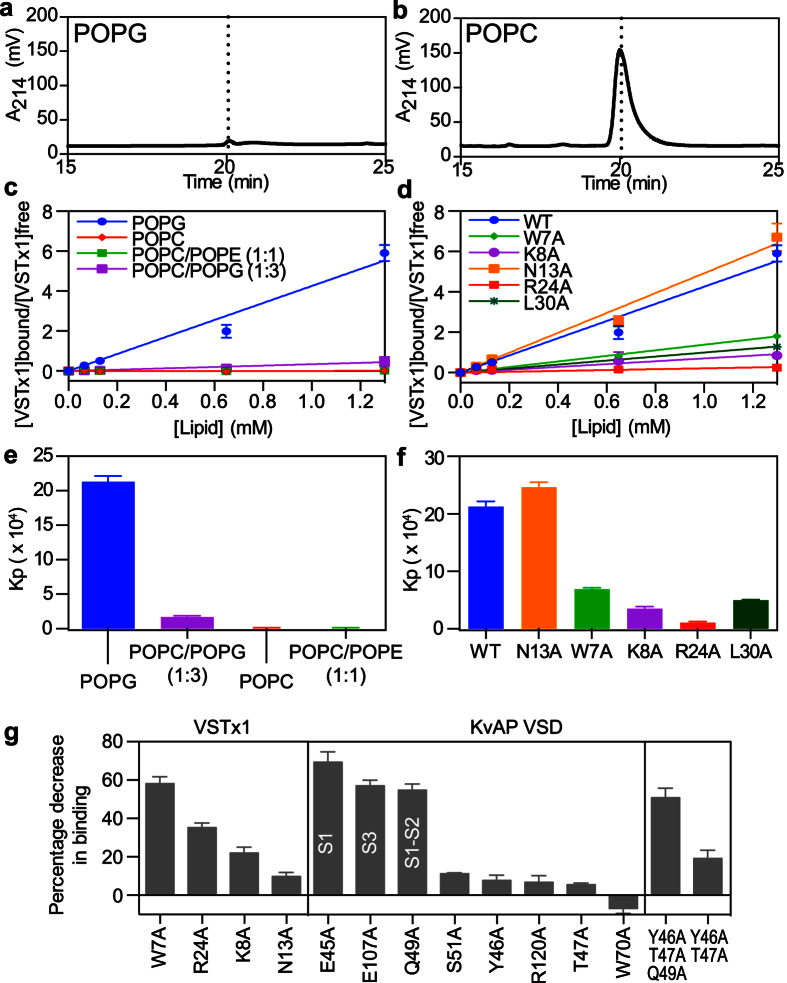
Lipid preference of VSTx1 and identification of key residues for lipid and VSD_K_ binding. RP-HPLC chromatograms showing the amount of VSTx1 present in the supernatant after ultracentrifugation with (**a**) 1.3 mM POPG or (**b**) 1.3 mM POPC liposomes. (**c**) Ratio of bound/free VSTx1 plotted as a function of lipid concentration using different lipids. (**d**) Ratio of bound/free VSTx1 for interaction of WT and mutant VSTx1 with POPG liposomes. (**e**) Partition coefficients (*K*_p_) for binding of VSTx1 to liposomes of different composition. (**f**) Partition coefficients (*K*_p_) for binding of WT and mutant VSTx1 to POPG liposomes. (**g**) Effect of mutations in VSTx1 and VSD_K_ on the avidity of the VSTx1-VSD_K_ interaction.

To investigate whether the micelle-binding interface identified by NMR-CSM faithfully reports on the lipid-binding interface of the peptide we used a previously reported liposome centrifugation assay^30^ to study the interaction between VSTx1 and phospholipids with different head groups. VSTx1 was incubated with liposomes of different composition, then the liposomes were pelleted using ultracentrifugation and the amount of unbound VSTx1 in the supernatant was determined using reverse-phase (RP) HPLC (Fig. 4a,b). Partition coefficients (*K*_p_) for binding of VSTx1 to liposomes were calculated by fitting to the partition equilibrium equation (see Eqn (3) in Materials and Methods). The *K*_p_ for binding to 1-palmitoyl-2-oleoyl-*sn*-glycero-3-phosphoglycerol (POPG) liposomes was 2.4 × 10^5^ while the *K*_p_ values for binding to POPC or 1-palmitoyl-2-oleoyl-*sn*-glycero-3-phosphoethanolamine (POPE) liposomes were close to 0 (both <10^2^) (Fig. 4e). Reducing the POPG content of liposomes by 25% reduced the *K*_p_ by 10-fold to 0.18 × 10^5^, suggesting that the peptide-lipid partition coefficient correlates with the negative charge density on the membrane surface, consistent with a previous report^30^. The close agreement with the previously published value indicates that the non-native N-terminal serine residue does not interfere with lipid binding.

To further examine the correlation between the NMR data (obtained with micelles) and the liposome centrifugation assay, we generated a panel of VSTx1 analogues where putative lipid-interacting residues identified in the NMR-CSM experiments (Trp7, Lys8, Arg24 and Leu30) were mutated to alanine. An N13A mutant was used as a negative control since it is located on the opposite face of VSTx1 to the hydrophobic patch and its chemical shift was not perturbed in the NMR-CSM experiments. Comparison of 2D ^1^H-^15^N HSQC spectra of each of the analogs with that of wild-type (WT) VSTx1 revealed that none of the mutations affected the global fold of the peptide. Chemical shift perturbations were limited to the residue altered by the mutation and nearest neighbor residues (Fig. S1).

The lipid-binding propensity of each mutant was examined by using the centrifugation assay with POPG liposomes (Fig. 4f). An R24A mutation nearly abolished partitioning into POPG liposomes, with *K*_p_ reduced by 95% (0.11 × 10^5^). W7A, K8A, and L30A mutants decreased *K*_p_ by 67%, 83%, and 76% respectively (to 0.77, 0.38 and 0.55 × 10^5^ respectively). As predicted, mutation of Asn13 to alanine did not cause a significant change in liposome binding (*K*_p_ = 2.7 × 10^5^). Our results are largely consistent with those previously reported for VSTx1 binding to lipid membranes^30^ and indicate that the hydrophobic patch and surrounding basic residues on VSTx1 mediate its partitioning into lipid membranes. The mutagenesis and NMR-CSM data are consistent, indicating that VSTx1 binds to micelles and liposomes in a similar manner. Thus, the apparent lack of VSTx1 interaction with POPC liposomes in the centrifugation assay presumably reflects the low affinity of the interaction rather than the complete absence of binding.

### The RW motif on VSTx1 is central to its interaction with KvAP

Since VSTx1 interacts with DHPC micelles, the interaction of the peptide with VSD_K_ solubilised in DHPC must be viewed as a three-component system. Thus, in order to delineate the residues that mediate the interaction of VSTx1 with VSD_K_, we performed NMR experiments in the presence of identical concentrations of micelles either in the presence or absence of the VSD_K_. A series of ^15^N-HSQC pairs (+/−VSD_K_) were acquired in which ^15^N-labeled VSTx1 was titrated with either unlabelled VSD_K_ purified in DHPC micelles or an equivalent concentration of DHPC micelles alone. Both chemical shift perturbations and peak broadening were observed in subsets of VSTx1 residues upon addition of VSD_K_ (Fig. 2b). A plot of the combined ^1^H/^15^N chemical shift perturbations (Δδ) shows that the chemical shifts of 16 backbone amides experienced perturbations higher than the cut-off value of one standard deviation of all perturbations (Fig. 3a). These residues are all located within the hydrophobic patch on VSTx1 and they overlap with the DHPC binding surface (Fig. 3b). Notably the chemical shift changes follow the same trajectory as seen for the empty micelles, indicating that the presence of the VSD increases the micelle binding affinity of the peptide (shifting the equilibrium closer to the bound state).

In addition to chemical shift changes, we observed severe broadening of a subset of the residues. More specifically, addition of 60 μM VSD_K_ to 20 μM VSTx1 led to complete loss of signal at the backbone amide NH of Arg24 and the sidechain NH of Trp25. A second group of resonances strongly affected by the binding were the backbone amides of Gly3, Lys10 and Phe34 as well as the sidechain NH of Trp27. The data series from the most severely affected residue (Arg24) was used to derive the dissociation constant (*K*_*d*_) for the interaction (see Eqn (2) in Materials and Methods). This yielded a *K*_*d*_ of 140 μM, which agrees well with the range of 100–500 μM previously derived from binding of VSTx1 to micelle-embedded KvAP immobilised on a Co^2+^ resin^34^.

In order to validate the NMR data, we used the panel of VSTx1 mutants generated for the liposome binding assay (W7A, K8A, N13A, R24A and L30A), and examined their ability to interact with VSD_K_ using a previously reported pulldown assay^27^. We note that the W25A and W27A VSTx1 constructs did not show over-expression, suggesting that these residues are important for the folding/stability of the peptide. In this assay, His_6_-tagged VSD_K_ micelles immobilized on nickel-NTA beads are used to capture VSTx1 (see Materials & Methods for details). Consistent with the NMR titration data, the W7A, K8A, and R24A mutants caused a 58%, 20%, and 38% decrease, respectively, in VSTx1 binding to VSD_K_ (Fig. 4g). Again, the N13A mutation (located distal to the hydrophobic patch) did not affect VSTx1 binding to VSD_K_.

### Both extracellular loops of the KvAP VSD are involved in binding VSTx1

NMR titrations were used to map the surface on VSD_K_ that mediates its interaction with VSTx1. ^2^H/^13^C/^15^N-labeled VSD_K_ was purified in DHPC micelles using a previously published protocol^22^. Backbone resonance assignments were obtained by acquiring transverse relaxation optimized spectroscopy (TROSY) 2D ^1^H-^15^N HSQC, 3D HNCO, and 3D HNCA spectra and matching the chemical shifts obtained to those reported previously for VSD_K_ (BioMagResBank accession code 16957)^23^.

**Figure 5:**
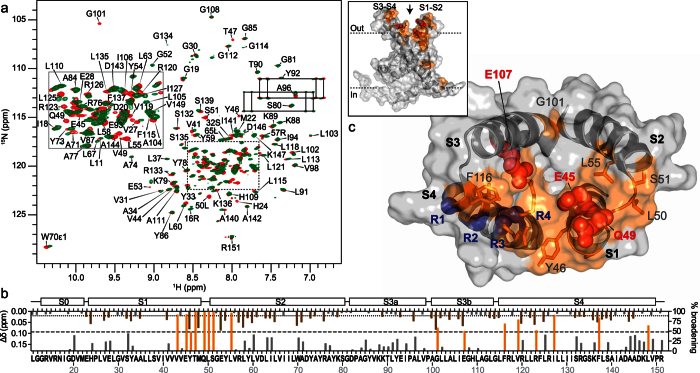
Identification of the VSTx1-binding surface of VSD_K_. (**a**) Overlay of 2D ^1^H-^15^N HSQC spectra of ^2^H/^13^C/^15^N-labeled VSD_K_ (100 μM) acquired in the absence (red) or presence (green) of 300 μM VSTx1. (**b**) Extent of peak broadening (%, right ordinate axis–orange/grey bars) and chemical shift perturbations (Δδ, left ordinate axis–brown bars) observed for VSD_K_ residues upon VSTx1 binding. Peak broadening was observed for residues in the S1-S2 and S3-S4 loops, indicating that both regions are involved in VSTx1 binding. (**c**) Molecular surface of VSD_K_ (PDB code 2KYH), viewed from the extracellular surface, with residues important for VSTx1 binding based on NMR-CSM and mutagenesis data highlighted in orange and red, respectively. The gating charges on S4 (R1, R2 and R3) are highlighted in blue.

2D ^1^H-^15^N HSQC and 3D HNCO spectra were acquired of ^2^H/^13^C/^15^N-labeled VSD_K_ in the absence or presence of unlabeled VSTx1 (Fig. 5a). The 3D HNCO spectra provided an extra chemical shift dimension for resolving resonance overlap in crowded spectral regions. The 3D HNCO data were acquired using non-uniform sampling in order to reduce acquisition time and increase resolution^37^. Peak broadening and chemical shift perturbations were observed for subsets of resonances upon addition of VSTx1 (Fig. 5b). Extensive chemical shift changes were observed throughout VSD_K_, consistent with the fast-exchange regime observed for binding of VSTx1 to micelles, which indirectly perturbs the chemical environment of VSD_K_ residues near the peptide-micelle interface. Peak broadening leading to significant loss of signal intensity at high VSTx1 concentrations was observed for 16 residues (V43, E45, Y46, T47, Q49, L50, S51, L55, G101, E107, F116, V119, R123, I127, F137, and L148). The majority of these residues (except F137 and L148) are located on the extracellular S1-S2 and S3-S4 loops of VSD_K_ and they all face a cleft formed between these two loops (Fig. 5c), identifying this as the putative peptide binding site.

To further pinpoint VSD_K_ residues involved in VSTx1 binding, residues in both the S1-S2 loop (E45, Y46, T47, Q49, S51) and S3-S4 paddle motif (E107, R120) were mutated to alanine. Far-UV circular dichroic (CD) spectra of these mutants overlaid well with that of WT VSD_K_ (Fig. S2), indicating that none of these mutations perturbed the global fold of the KvAP voltage sensor. The ability of each of these VSD_K_ analogs to interact with VSTx1 was then examined using the pulldown assay described above. Of the seven mutations, E45A and Q49A on helix S1 and E107A on helix S3 caused a 65%, 58%, and 59% decrease, respectively, in VSD_K_ binding to VSTx1 (Fig. 4g). Taken together with the NMR-CSM data, these mutations provide further evidence that the peptide binds to residues located inside the extracellular-facing cleft formed between the four transmembrane helices of VSD_K_.

**Figure 6:**
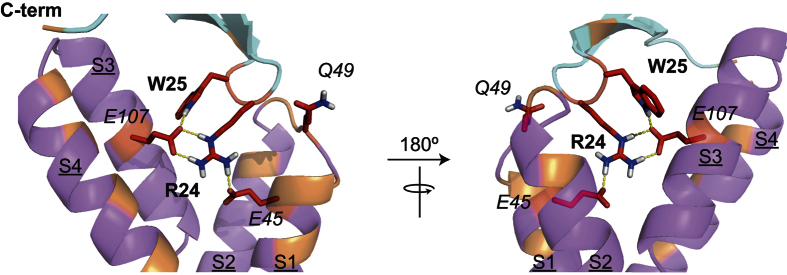
Docking model of the VSD_K_-VSTx1 complex. Residues implicated by the NMR data as being involved at the toxin-channel interface are highlighted in red (toxin residues) and orange (channel residues). VSD_K_ residues confirmed to be at the binding interface by mutagenesis are shown in red (E45/Q49/107), whilst peptide residues shown in red (R24/W25) are those that showed the greatest perturbations in NMR titrations with VSD_K_. Orange residues are the remaining residues indicated by the titrations to be perturbed by complex formation. Hydrogen bonds are shown as yellow dotted lines. The helices are marked as S1-S4 in underlined font, while channel residues are in italics and peptide residues are in bold font. The C-terminus of the peptide is indicated near the S3-S4 loop.

The titration revealed residues on the peptide and on the VSD_K_ that showed significant line broadening upon titration. These residues were used to derive a model of the complex using HADDOCK docking software^38^. The program automatically randomizes protein orientations, where the two partner proteins are positioned at 150 Å from each other in space and each protein is randomly rotated around its center of mass. The titration identified two clusters of surface exposed residues that were severely (Arg24, Trp25) and moderately broadened (Lys10, Trp27 and Phe34). These were included as active and passive residues, respectively, for the docking. VSD_K_ residues that showed reduced binding in our mutagenesis studies (E45, Q49, E107) were identified as active residues. The remaining surface-exposed residues in the extracellular loops that showed significant peak broadening in the NMR experiments (V43, Y46, T47, L50, S51, L55, F116, V119, R123, I127) were included as passive residues. HADDOCK identified three clusters of VSTx1-VSD_K_ complexes. The most populated and lowest-energy cluster showed binding of VSTx1 residues Arg24 and Trp25 to VSD_K_ residues E45 and E107 whilst peptide residue Phe34 was found proximal to residues F116 and R123 of VSD_K_ (Fig. 6–PDB file of this model is included as Supplemental Data 1).

## Discussion

VSTx1 was isolated from the venom of the Chilean rose tarantula *Grammostola rosea* (previously *G. spatulata)* over a decade ago^27^. It partitions into lipid membranes^33^ and has been used as a model for studying lipid-protein interactions^36^. We used NMR-CSM to show that a hydrophobic patch containing tryptophan residues from loops 1 and 4 mediates binding of VSTx1 to lipid micelles (Fig. 3a). We also established that this binding mode is likely conserved in liposomes by performing a well-established centrifugation assay using a number of VSTx1 mutants. Our combined results are consistent with previous studies showing that the tryptophan residues in VSTx1 are exposed to a more hydrophobic environment upon membrane binding^13^,^30^,^39^. These results contrast with a recently published VSTx1 lipid-binding model based on neutron diffraction data in which Trp7 and Gln13 are found together at the lipid interface^33^. We found that only Trp7 makes important interactions with DHPC micelles, while residues Lys10 to Asp18 (loop 2) face away from the lipid-binding interface^33^. We also showed that the VSTx1 interaction with POPG liposomes was nearly abolished by K8A or R24A mutations (Fig. 4f). We conclude that binding of VSTx1 to lipid membranes containing acidic phospholipids is mediated by the hydrophobic patch and proximal cationic residues.

The presence of VSD_K_ enhances the micelle binding of VSTx1, but the NMR data indicates that the binding pose of the peptide is conserved in the presence of the VSD_K_. In addition, we observed significant broadening of specific VSTx1 resonances that were indicative of a higher affinity binding event (than observed for micelles alone). The residues most significantly perturbed by the presence of the VSD_K_ were Arg24 and Trp25, with the binding constant of these independently quantified to be in the μM range. Thus, VSTx1 appears to bind specifically, but weakly, to phospholipid membranes, whereas binding to the VSD_K_ is both specific and moderately potent.

NMR titrations were also used to identify residues on VSD_K_ that mediate its interaction with VSTx1. Both peak broadening and chemical shift perturbations were observed upon titration of VSD_K_ with VSTx1 (Fig. 5b). The chemical shift changes are indicative of interactions in the fast-exchange regime and consistent with transient binding of the peptide to the detergent micelles containing the VSD_K_ rather than specific binding to the VSD_K_ itself. These perturbations mimic those seen for VSTx1 binding to the micelles and were unsurprisingly observed across the entire VSD_K_.

In the VSTx1 titration we observed severe broadening of a subset of residues due to the presence of the VSD_K_. Since the reaction kinetics will be conserved regardless of which binding partner is observed, we would expect that the opposite titration would reveal a subset of residues in VSD_K_ that undergo severe broadening upon VSTx1 addition. Indeed, this was borne out in our experiments. Most of the amide groups that suffered significant peak broadening (>50% of average broadening) were from residues located in a pocket formed between the S1-S2 and S3-S4 extracellular loops (Fig. 5c), identifying this as the putative VSTx1-VSD_K_ interface. We also note that a number of residues experience weak-to-moderate broadening (<50% of average broadening), indicating the presence of an exchange process on a similar timescale. This exchange regime is consistent with conformational exchange on the μs-ms time scale. Taken together, the data suggest that the presence of the peptide also induces a small but global conformational change in VSD_K_.

During the gating cycle, KvAP has been proposed to transition from the closed “down” state of the channel to a pre-activated state, at which point it can either transition to the activated open “up” state or an inactivated “up” state. These transitions are driven by conformational changes of the VSD in response to changes in the membrane voltage. VSTx1 has been shown to bind to the inactivated state of KvAP^28^. In our experiments VSD_K_ does not experience an electric field and it is reasonable to assume that it adopts the “up” configuration. This conformation may either be consistent with the open or the inactivated state of the channel, which indeed may or may not represent different VSD_K_ conformational states. The conformational change in VSD_K_ that we observed upon VSTx1 binding suggests that the peptide stabilizes a state different to that found in the absence of the peptide. This can most simply be interpreted as stabilization of the inactivated state from some other stable conformation in lipid micelles. This also suggests that the experimentally determined structure of VSD_K_ in micelles^22^,^23^ is similar to but not identical to the inactivated state. NMR relaxation studies of VSD_K_ revealed considerable motion in the core of the protein occurring on the μs-ms timescale, indicating a degree of inherent structural plasticity^23^. We note in particular that our binding studies identify G101 in the core of the protein as being broadened by VSTx1 binding, and that the previous relaxation studies found this residue to be particularly prone to exchange broadening in both micelles and lipid nano-discs. It is therefore reasonable to conclude that VSTx1 stabilises a conformational state that is present but not observed directly in previous NMR studies. Thus, our data support a model where there are two distinct “up” conformations, corresponding to the inactivated and the open states of the channel. We propose that the solution structure is of the open “up” state and that the conformational changes we observed upon VSTx1 binding would transition the VSD_K_ to the previously unobservable inactivated “up” state.

Site-directed mutagenesis of both VSTx1 and VSD_K_ was used to confirm and extend the conclusions drawn from the NMR data. W7A, K8A and R24A mutations significantly reduced VSTx1 binding to KvAP micelles, whereas an N13A mutation did not, consistent with the NMR data (Fig. 3). The difficulty in producing the critical W25A mutation and the overlap between micelle and VSD_K_ binding of R24 did not permit us to conclusively confirm the NMR-CSM data through VSTx1 mutagenesis. Of the nine VSD_K_ mutants we generated, only mutation of residues E45, Q49, and E107 perturbed the interaction with VSTx1 (Fig. 4g). E45 and Q49 are located in the S1-S2 loop while E107 is located on S3 in the S3-S4 loop. All of the residues are located in the aqueous cleft formed between the S1-S2 and S3-S4 loops (Fig. 5c), suggesting that VSTx1 binds inside this cleft. This peptide binding interface is largely consistent with recent NMR-based cross-saturation transfer experiments^13^, however, our NMR-CSM data as well as the mutagenesis data suggest an alternative position of the peptide with respect to the channel. The NMR cross-saturation experiments performed on VSD_K_, which were based on non-specific isotope labeling, position the peptide outside the vestibule, formed between the four helices, facing the crevice between the S1 and S4 helices, (i.e. not making contact with E107 on S3). The data presented shows that residues on the S2 and S3 helices are also involved in the interaction, placing toxin residues within the vestibule.

**Figure 7:**
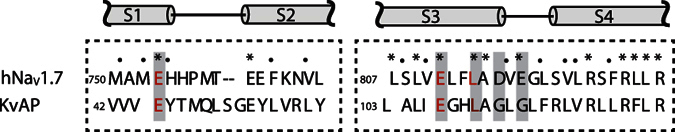
Sequence alignment of VSD_K_ with the domain II VSD of Na_V_1.7. Conserved residues are indicated with filled circles while identical residues are highlighted with an asterisk. Residues highlighted in grey boxes are those previously established to confer sensitivity of Na_V_1.7 to the gating modifier peptide HwTx-IV. Conserved residues that have been shown to confer sensitivity of VSD_K_ or Na_V_1.7 to gating modifier peptides are highlighted in red.

Several reports have revealed the involvement of the S1-S2 loop in binding of this class of peptides to voltage-gated ion channels. In particular we find good agreement between our data and comprehensive mutagenesis studies that identified the binding site on Na_V_1.7 for the related gating modifier toxin HwTx-IV^40^. Mutagenesis of the Na_V_1.7 domain II VSD revealed five residues in the S1-S2 and S3-S4 loops that are critical for channel inhibition by HwTx-IV. Three of these five residues are conserved in KvAP (see sequence alignment in Fig. 7). Remarkably, two of these residues (E45/E107 in KvAP *vs* E753/E811 in Na_V_1.7) were found by mutagenesis to be important determinants of the sensitivity of Na_V_1.7 and VSD_K_ to gating modifier peptides^36^. Target promiscuity has previously been reported for this class of peptides, and it is striking that VSTx1 inhibits Na_V_1.7 with moderate potency (IC_50_ ~ 4 μM). We note that the pharmacophore of the Na_V_1.7 inhibitor HwTx-IV has been established by comprehensive mutagenesis studies to contain two critical residues, Trp30 and Lys32^18^,^19^, where the Trp and Lys sidechains assume a similar spatial arrangement to that found here for VSTx1 residues Arg24 and Trp25^17^. This provides compelling evidence that this class of spider-venom peptides bind to the VSD of VGICs via a conserved molecular mechanism.

To provide a molecular model of the VSTx1–VSD_K_ complex we used our atomic-resolution NMR data supplemented with mutagenesis results. We performed (semi)-rigid body docking using ambiguous restraints in the HADDOCK program. We caution that our data predicts that the peptide-bound VSD_K_ structure will be close to but not identical to the previously solved crystal/NMR structures. Nevertheless, construction of such a model is useful for visualizing and interpreting the data. The 3D structure of the complex consistently showed a pose where Arg24 is sandwiched between E45 and E107 (equivalent to Na_V_1.7 residues E753 and E811) whilst the indole group of Trp25 makes contact with E107. There are a total of four hydrogen bonds (<2 Å) between these residues. In the docking models the C-terminus of the peptide appears to make contact with the S3-S4 loop, where Phe34 is in some poses positioned between R116 and R119 and in other cases the Phe34 aromatic ring is π-stacked with channel residue F116. The remaining loop 4 residues of VSTx1 sit against the S1-S2 loop. With the exception of the sidechain amide carbonyl of Q49, which in some docking poses makes contact with the Arg24 backbone amide NH, no specific interactions were consistently observed between loop 4 residues of VSTx1 and the S1-S2 loop of VSD_K_. The orientation of the peptide with respect to the VSD places a hydrophobic patch comprising residues Gly3/Met6/Trp7/Lys8 facing the S2-S3 cavity and another hydrophobic patch comprising residues Leu19/Val20/Ser22/Leu30/Ala31/Ser32/Phe34 facing the S1-S4 cavity (see also PDB file in Supplemental Data 1). We note that although our docking model does not contain any lipids, these residues were independently identified as lipid-facing by the NMR titrations, further supporting the docking model.

Previous studies of the interaction between VGICs and gating modifier peptides have focused on interactions with the S3-S4 voltage-sensor paddle^3^,^41^,^42^. In particular, a number of studies that employed chimeric and mutant channels concluded that gating modifier peptides interact primarily with the S3-S4 paddle region^42^,^43^. In this study we support the growing literature pointing to involvement of the S1-S2 loop as an important component in binding of gating modifiers^40^,^42^,^44^,^45^. Given the relatively large footprint of venom peptides, it is perhaps not surprising that these peptides can contact both the S3-S4 paddle and the S1-S2 loop.

## Conclusions

The structures of K_V_ channels and bacterial Na_V_ channels reveal a significant aqueous cleft between the S1-S2 and S3-S4 loops. In the Na_V_Ab channel, this cleft extends ~10 Å into the membrane^46^. Our data suggest that gating modifier peptides bind within this aqueous cleft and make specific contacts with anionic residues on S1 and S3 that face into the cleft (Fig. 6), thereby disrupting intramolecular interactions between counter charges to the cationic S4 gating charges. The hydrophobic loop 1 and C-terminal residues of the peptide maintain binding to the two lipid-exposed sides of the cleft. The resulting network of lipid-peptide-channel interactions increases the energetic barrier to the conformational changes required for channel gating and we propose that this is the mechanism by which gating modifier toxins inhibit VGICs. Our results point to a conserved mechanism of channel binding amongst gating modifier peptides and provide a template for engineering of peptides with improved potency and selectivity for use as drugs and insecticides.

## Methods

### VSTx1 sample preparation

A codon-optimised synthetic gene encoding VSTx1 was synthesised by GeneArt and subcloned into a modified version of the pLIC expression vector^29^. Competent *E. coli* BL21 cells transformed with the expression plasmid were grown in LB broth at 37 °C until OD_600_ ~ 0.2, then the growth temperature was reduced to 16 °C. Protein production was induced at OD_600_ ~ 1 by addition of IPTG to a final concentration of 0.2 mM and allowed to proceed overnight at 16 °C. For production of ^13^C/^15^N-labelled peptide, LB broth was replaced with M9 minimal medium supplemented with ^13^C D-glucose and ^15^NH_4_Cl. Cells were harvested by centrifugation for 15 min at 4 °C and 5,000 *g*.

Cells overexpressing a His_6_-maltose-binding protein (MBP)-VSTx1 fusion protein were resuspended in equilibration buffer (400 mM NaCl, 40 mM Tris pH 8.0) and lysed by continuous flow cell disruption (TS Series Benchtop System, Constant Systems Ltd) at a constant pressure of 26 kpsi. The MBP-VSTx1 fusion protein was purified over a Ni-NTA column in the same buffer. Protein was eluted with equilibration buffer supplemented with 250 mM imidazole, and desalted using a Amicon Ultra-15 concentrator. MBP was cleaved from the peptide using 50 μg/ml TEV protease in buffer containing 3 mM GSH and 0.3 mM GSSG for 18 h at room temperature. Cleaved peptide was RP-HPLC, using a solvent system where Solvent A is water with 0.05% TFA, and Solvent B is ACN with 0.043% TFA, at a flow rate of 3 ml/min and a gradient going from 20–60% solvent B over 35 minutes over a C_4_ column (Phenomenex Jupiter, 10 μ, C_4_, 300A, 250 × 10 mm). The yield of purified peptide was 1.5 mg per litre of culture. Peptide purity was assessed using analytical RP-HPLC and electrospray ionisation mass spectrometry (ESI-MS), showing a mass of 4084.4, in good agreement with the calculated oxidised mass of 4084.8.

### VSD_K_ preparation

A codon-optimized synthetic gene encoding VSD_K_ was synthesized by GeneArt and subcloned into a modified version of the pET-19b expression vector. For production of ^2^H/^13^C/^15^N-labelled VSD_K_, BL21-RIPL (Stratagene) cells transformed with the VSD_K_ expression vector were grown in 5 ml 33% ^2^H_2_O LB broth overnight at 37 °C. Cells were pelleted by centrifugation for 10 min at 3,000 *g* and resuspended in 1 L 67% ^2^H_2_O M9 minimal medium. The culture was shaken at 37 °C until OD_600_ ~ 0.8, then cells were pelleted by centrifugation for 10 min at 3,000 *g* and resuspended in 1 L 99% ^2^H _2_O M9 minimal medium. Cells were grown at 37 °C for 1 h before expression was induced by addition of IPTG to a final concentration of 0.2 mM and allowed to proceed 3 h at 37 °C.

VSD_K_ was purified as described previously^22^. Briefly, harvested cells were lysed and purified over TALON resin in purification buffer (25 mM Tris pH 8, 100 mM KCl, 0.26% *n*-decyl-β-D-maltopyranoside (DM)) and subsequently exchanged into the final buffer (20 mM 4-(2-hydroxyethyl)-1-piperazine ethanesulfonic acid (HEPES) pH 7.0, 20 mM KCl, and 5 mM 1,2-diheptanoyl-*sn*-glycero-3-phosphocholine (DHPC)) by size exclusion chromatography using a Superdex 200 column. The fraction with the highest VSD_K_ concentration (0.12 mM) was used directly for NMR experiments. The final yields of purified VSD_K_ was ~5 mg and ~2 mg per litre of bacterial culture for unlabelled and ^2^H/^13^C/^15^N-labelled VSD_K_ respectively. Far-UV CD spectra were measured in cuvettes having an optical path length of 0.1 mm, at room temperature. Samples in size exclusion chromatography buffer were used for analysis.

### NMR data acquisition and calculation of VSTx1 structure

Lyophilised VSTx1 was dissolved at a final concentration of 1.7 mM in 20 mM sodium acetate buffer pH 4.5 containing 5% ^2^H_2_O. NMR spectra were recorded at 25 °C using a Bruker 900 MHz spectrometer equipped with a cryogenically cooled probe. All spectra acquired for making backbone and sidechain resonance assignments were collected using non-uniform sampling (NUS) in order to expedite data acquisition^47^, and processed using MaxEnt^48^. In addition, NUS was used to acquire a long-range HNCO experiment designed to detect through-hydrogen-bond scalar couplings and a 4D HCC(CO)NH-TOCSY experiment used for sidechain assignment^49^. 3D ^13^C- and ^15^N-edited NOESY-HSQC spectra were acquired using linear sampling. All spectra were analysed using XEasy^50^. Backbone ϕ and ψ dihedral angle restraints were generated from chemical shifts using the program TALOS-N^32^. Dihedral-angle restraints were used together with estimates of interproton distances from NOESY spectra, hydrogen-bond constraints from the long-range HNCO experiment, and disulfide-bond restraints to calculate the structure of VSTx1 using CYANA 3.0^51^.

### NMR-CSM experiments

For mapping the lipid interaction surface of VSTx1, we acquired HSQC spectra at 25 °C of 30 μM ^15^N-labelled VSTx1 in buffer containing 20 mM HEPES pH 7, 5% ^2^H_2_O, and 0–1% (w/v) DHPC (0.1%, 0.2%, 0.5% and 1%). For mapping the VSD_K_ interaction surface on VSTx1, we acquired HSQC spectra at 25 °C of 20 μM ^15^N-labelled VSTx1 in buffer containing 20 mM HEPES pH 7, 20 mM KCl, 0.2% DHPC, 5% ^2^H_2_O, and with 0–100 μM VSD_K_ (10 μM, 20 μM, 60 μM and 100 μM). The combined chemical shift changes (Δδ) were calculated using Eqn (1):


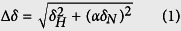


where δ_H_ and δ_N_ are the chemical shift perturbations in the ^1^H and ^15^N dimensions, respectively, and α is a ^15^N chemical shift scaling factor (0.15). For each series of experiments, the standard deviation of all chemical shift changes was calculated and used as the cut-off value for determining significance^52^. For mapping the VSTx1 interaction surface on VSD_K_, HSQC spectra were acquired of 100 μM ^2^H/^13^C/^15^N-labelled VSD_K_ with 0, 100, and 300 μM of VSTx1. Spectra were recorded at 42 °C for the VSTx1/VSD_K_ titration. The binding constants were derived from Eqn 2 as previously described^52^:


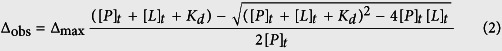


where, Δ_obs_ is the change in the observed signal intensity from the free state, Δ_max_ is the maximum change in intensity, and *P* and *L* refer to VSTx1 and VSD_K_ respectively.

### VSTx1 centrifugation assay

Large unilamellar vesicles (LUVs) were prepared by drying lipids (POPG and/or POPC, 25 mg/mL) under nitrogen gas, drying the lipid pellet under vacuum, and resuspending the lipids by vortexing for 1 h in an aqueous buffer containing 10 mM HEPES pH 7.4, and 150 mM NaCl. Vesicles were extruded 20 times using 0.1 μm polycarbonate filters (Avanti Polar Lipids, Inc.). Aqueous solutions of the toxin (10 μM) were incubated with varying amounts of vesicles for 1 h at room temperature, then the vesicles were centrifuged for 30 min at 100,000 *g* before the toxin concentration in the aqueous phase was determined. Aqueous concentrations of VSTx1 were quantified by RP-HPLC using a monolithic C_18_ column (4.6 × 100 mm). The toxin was eluted with a linear gradient of 10–50% solvent B (0.046% TFA in acetonitrile) in solvent A (0.05% TFA in water) over 10 min at a flow rate of 3 ml/min. The partition coefficients (*K*_p_) for the binding of VSTx1 to liposomes were calculated by fitting to the partition equilibrium equation:


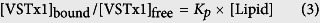


### VSTx1 pulldown assay

VSD_K_ was purified in *n*-dodecyl-β-D-maltopyranoside (DDM) as described above, and 50 μl of Ni-NTA resin was then saturated with the purified VSD_K_. VSTx1 was dissolved at a concentration of 100 μM in the same buffer used to purify VSD_K_ (25 mM Tris pH 7.5, 100 mM KCl, 0.05% DDM). VSTx1 solution (50 μl) was added to the resin and the mixture was then incubated for 30 min (whilst rolling). The resin was washed with two column volumes of the same buffer, then any protein remaining on the column was eluted with buffer containing 250 mM imidazole. The eluted protein was reduced by addition of DDT to 50 mM and then incubated at 37 °C for 2 h to improve separation. The reduced sample was analysed by RP-HPLC using an analytical C_18_ column.

## Additional Information

**Accession codes**: 2N1N. VSTx1 BMRB accession code: 25568.

**How to cite this article**: Lau, C. H. Y. *et al*. Molecular basis of the interaction between gating modifier spider toxins and the voltage sensor of voltage-gated ion channels. *Sci. Rep.* **6**, 34333; doi: 10.1038/srep34333 (2016).

## Supplementary Material

Supplementary Information

## Figures and Tables

**Table 1:** Structural statistics for the ensemble of VSTx1 structures.

Experimental restraints^1^	
Interproton distance restraints	
Intraresidue	193
Sequential (|i–j| = 1)	206
Medium range (|i–j| < 5)	116
Long range (|i–j| ≥ 5)	179
Disulfide-bond restraints	9
Dihedral-angle restraints (ϕ, ψ, χ_1_)	54
Total number of restraints per residue	22.5
R.m.s. deviation from mean coordinate structure (Å)^2^	
Backbone atoms, residues 2–34	0.03 ± 0.01
Heavy atoms, residues 2–34	0.54 ± 0.11
Stereochemical quality^3^	
Residues in most favored Ramachandran region (%)	89.8 ± 1.7
Ramachandran outliers (%)	0.0 ± 0.0
Unfavorable sidechain rotamers (%)	15.2 ± 4.6
Clashscore, all atoms^4^	5.8 ± 0.7
Overall MolProbity score	2.72 ± 0.14

All statistics are given as mean ± S.D. ^1^Only structurally relevant restraints, as defined by CYANA, are included.

^2^Mean r.m.s. deviation calculated over the entire ensemble of 20 structures.

^3^According to MolProbity (http://molprobity.biochem.duke.edu).

^4^Defined as the number of steric overlaps >0.4 Å per thousand atoms.
