# Supplementary material for: Molecular basis of the interaction between gating modifier spider toxins and the voltage sensor of voltage-gated ion channels
Source: Sci Rep. 2016 Sep 28;6:34333. doi: 10.1038/srep34333 (PMC5039624; doi:10.1038/srep34333)
Supplement: Supplementary Information [file srep34333-s1.pdf]

Molecular basis of the interaction between gating modifier spider toxins and the voltage sensor of voltage-gated ion channels

Carus H.Y. Lau[1], Glenn F. King[1,*] and Mehdi Mobli[2,*]

[1]Institute for Molecular Bioscience and [2]Centre for Advanced Imaging,

The University of Queensland, St. Lucia, QLD 4072, Australia

***Address for correspondence:** m.mobli@uq.edu.au or glenn.king@imb.uq.edu.au

Supplementary Information

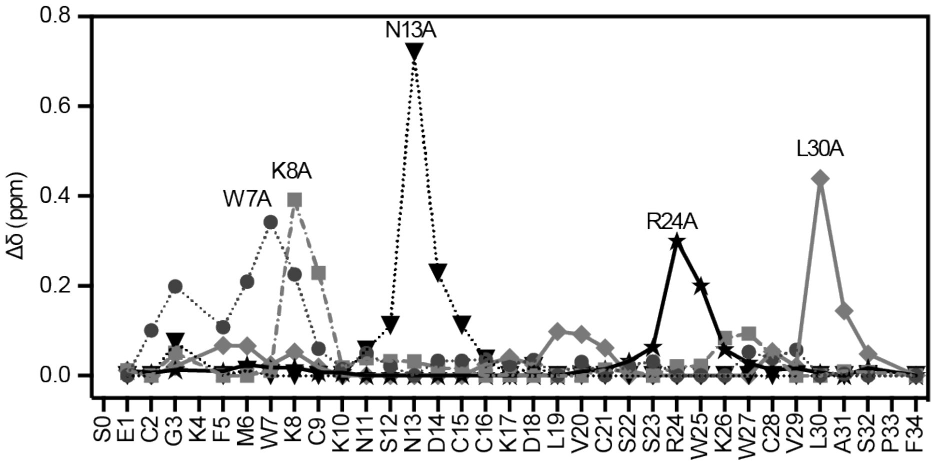

Figure S1. Combined chemical shift difference for individual residues of VSTx1 compared to the wild-type toxin. Chemical shift perturbations were limited to the residue altered by the mutation and nearest neighbor residues. None of the mutations perturb the global fold of VSTx1.

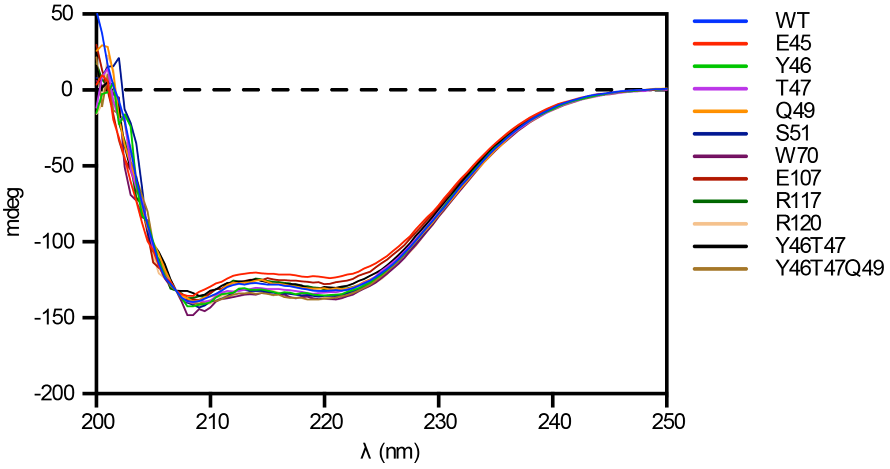

Figure S2. Far-UV circular dichroic (CD) spectra of KvAP mutants. All spectra overlaid well with that of wild-type VSD_K indicating that all mutants have a similar global fold to the native KvAP voltage sensor.
